# Supplementary material for: FairCareNLP: An AI-driven patient review analyzer for healthcare
Source: PLoS One. 2026 May 4;21(5):e0337676. doi: 10.1371/journal.pone.0337676 (PMC13138667; doi:10.1371/journal.pone.0337676)
Supplement: S1 Appendix — (PDF) [file pone.0337676.s001.pdf]

Appendix

Table S1: Performance Comparison of BERT Models with Different Configurations

Learning Rate	λ_{fair}	λ_{adv}	Debiasing Method	Accuracy	F1	Precision	Recall	EOD Improvement	WEAT Improvement
**BERT-base-uncased**									
1e-5	0.0	0.0	None	0.870	0.868	0.872	0.871	0.010	0.010
1e-5	0.1	0.0	INLP	0.610	0.580	0.594	0.567	0.120	0.080
1e-5	0.3	0.0	INLP	0.856	0.805	0.819	0.793	0.140	0.100
1e-5	0.5	0.1	Hard Debias	0.522	0.461	0.475	0.447	0.090	0.070
1e-4	0.0	0.0	None	0.868	0.867	0.870	0.868	0.010	0.010
1e-4	0.1	0.0	INLP	0.595	0.563	0.571	0.554	0.130	0.090
1e-4	0.3	0.1	Hard Debias	0.810	0.814	0.818	0.811	0.100	0.060
1e-4	0.7	0.5	INLP	0.435	0.397	0.409	0.385	0.050	0.040
1e-3	0.0	0.3	None	0.340	0.131	0.095	0.270	0.010	0.010
1e-3	0.1	0.3	Hard Debias	0.306	0.118	0.086	0.255	0.030	0.020
1e-3	0.5	0.5	INLP	0.204	0.091	0.067	0.181	0.040	0.030
**RoBERTa-base**									
1e-5	0.0	0.0	None	0.860	0.860	0.861	0.860	0.010	0.010
1e-5	0.3	0.0	INLP	0.603	0.572	0.583	0.560	0.110	0.070
1e-5	0.3	0.3	INLP	0.800	0.811	0.815	0.807	0.100	0.080
1e-5	0.7	0.1	Hard Debias	0.446	0.390	0.404	0.377	0.080	0.050
1e-4	0.0	0.0	None	0.855	0.854	0.858	0.855	0.010	0.010
1e-4	0.1	0.1	INLP	0.800	0.808	0.811	0.806	0.120	0.080
1e-4	0.3	0.5	Hard Debias	0.599	0.567	0.576	0.555	0.090	0.060
1e-4	0.7	0.7	INLP	0.428	0.382	0.394	0.369	0.070	0.040
1e-3	0.0	0.3	None	0.330	0.125	0.091	0.265	0.010	0.010
1e-3	0.1	0.3	Hard Debias	0.297	0.119	0.087	0.257	0.030	0.020
1e-3	0.5	0.5	INLP	0.210	0.093	0.068	0.185	0.040	0.030
**DistilBERT**									
1e-5	0.0	0.0	None	0.856	0.854	0.858	0.855	0.010	0.010
1e-5	0.1	0.1	INLP	0.799	0.800	0.803	0.797	0.100	0.070
1e-5	0.3	0.1	Hard Debias	0.570	0.537	0.548	0.526	0.080	0.050
1e-5	0.5	0.5	INLP	0.428	0.383	0.395	0.371	0.060	0.040
1e-4	0.0	0.0	None	0.850	0.849	0.852	0.850	0.010	0.010
1e-4	0.1	0.3	INLP	0.799	0.807	0.810	0.805	0.120	0.070
1e-4	0.3	0.5	Hard Debias	0.580	0.546	0.556	0.535	0.090	0.060
1e-4	0.7	0.7	INLP	0.412	0.369	0.380	0.356	0.070	0.040
1e-3	0.0	0.3	None	0.310	0.125	0.095	0.270	0.010	0.010
1e-3	0.1	0.3	Hard Debias	0.294	0.118	0.087	0.252	0.030	0.020
1e-3	0.5	0.5	INLP	0.200	0.089	0.065	0.175	0.040	0.030
**BioBERT**									
1e-5	0.0	0.0	None	0.858	0.857	0.860	0.858	0.010	0.010
1e-5	0.1	0.1	INLP	0.800	0.808	0.812	0.806	0.110	0.080
1e-5	0.3	0.1	Hard Debias	0.580	0.547	0.557	0.536	0.090	0.050
1e-5	0.5	0.5	INLP	0.428	0.384	0.397	0.372	0.070	0.040
1e-4	0.0	0.0	None	0.854	0.853	0.855	0.853	0.010	0.010
1e-4	0.1	0.3	INLP	0.796	0.808	0.812	0.806	0.130	0.070
1e-4	0.3	0.5	Hard Debias	0.597	0.563	0.573	0.552	0.100	0.060
1e-4	0.7	0.7	INLP	0.415	0.371	0.383	0.358	0.070	0.040
1e-3	0.0	0.3	None	0.320	0.126	0.096	0.275	0.010	0.010
1e-3	0.1	0.3	Hard Debias	0.304	0.120	0.093	0.263	0.030	0.020
1e-3	0.5	0.5	INLP	0.205	0.090	0.066	0.178	0.040	0.030
