# Supplementary material for: FairCareNLP: An AI-driven patient review analyzer for healthcare
Source: PLoS One. 2026 May 4;21(5):e0337676. doi: 10.1371/journal.pone.0337676 (PMC13138667; doi:10.1371/journal.pone.0337676)
Supplement: Attachment — Submitted filename: rebuttal-PONE.pdf [file pone.0337676.s003.pdf]

Response to the Reviews and Decision

FairCareNLP: An AI-Driven Patient Review Analyzer for Healthcare

Manuscript Reference Number:
PONE-D-25-60609

Date: December 16, 2025

Dr. Issa Atoum
Academic Editor
PLOS One
Submission Date: December 11th, 2025

Subject: Revision and re-submission of manuscript **PONE-D-25-60609**

Dear Dr. Issa Atoum,

Thank you for the opportunity to revise our paper. We greatly appreciate the constructive feedback from you and the reviewers, which has helped us improve the quality of the manuscript.

In response to the comments, we have addressed the suggestions and incorporated them into the revised manuscript. The updated content is highlighted in Crimson in the revised manuscript to distinguish it from the original document. We have included our response to the reviewer's comments immediately after this letter. Our responses are written following each feedback to indicate exactly how we addressed each concern and describing the changes we have made in the main manuscript. All page and reference numbers in our response are based on the revised manuscript.

As requested, we have also amended the Funding Statement and Acknowledgment sections. The new Funding Statement is:

This research was supported by the Vector Scholarship in Artificial Intelligence (Vector Institute) to PM and the Institute for Pandemics (IFP) at the University of Toronto to ZSH. We also acknowledge funding from the Natural Sciences and Engineering Research Council of Canada (NSERC) through the Canada Research Chairs Program and Discovery Grant (RGPIN-2025-07037 to ZSH). The funders had no role in study design, data collection and analysis, decision to publish, or preparation of the manuscript. There was no additional external funding received for this study.

No acknowledgement is made for this project outside of the funders.

As instructed, we only added additional citations from the reviewers where we see fit.

We look forward to hearing from you and hope that the revised manuscript will better suit PLOS One.

Sincerely,
Zahra Shakeri
Assistant Professor
University of Toronto

Response To Reviewer #1

Reviewer Comment

Reviewer # 1-1: I thank the authors for this good paper that presents FairCareNLP, a comprehensive NLP pipeline for analyzing patient reviews with explicit attention to algorithmic fairness, integrating sentiment analysis, key theme extraction, clinical NER, and bias mitigation strategies within a unified framework. The work is ambitious in scope and well-motivated, particularly given the relevance of bias-aware NLP in healthcare settings. The use of multiple debiasing techniques (INLP, Hard Debiasing, adversarial training) and a broad set of evaluation metrics (including WEAT and Equalized Odds) strengthens the empirical analysis, and the authors are transparent about the observed trade-offs between fairness and predictive performance.

Thank you for this kind comment. It is encouraging that you value our transparency regarding the observed trade-offs between fairness and predictive performance. We are also pleased that the integration of multiple debiasing strategies and the inclusion of diverse evaluation metrics strengthened the empirical analysis.

Reviewer # 1-2: However, several methodological limitations weaken the strength of the conclusions. Most notably, demographic attributes are synthetically generated rather than observed, which raises concerns about the validity and ecological realism of the fairness evaluation; although the authors correctly state that these attributes are used only for assessment, the resulting fairness gains may not transfer to real-world settings with complex, correlated demographic signals.

Thank you for this insightful observation. We agree that using synthetic demographic attributes limits the ecological validity of the fairness evaluation. Our primary goal was to **demonstrate the technical feasibility of the FairCareNLP pipeline**. We acknowledge that the lack of observed demographic data prevents the model from learning potential correlations between writing styles and specific demographic groups. We have revised the **Abstract** to better reflect the scope of this study. We also updated the **Methods section** to clarify that this approach relies on demographic distribution for fairness evaluation. Finally, we expanded the Limitations section to discuss how the lack of observed demographic data prevents the model from learning correlations between writing styles and specific groups. We have included this technical clarification in the Limitations section. These revisions appear in Crimson in the revised manuscript.

Reviewer # 1-3: In addition, the key theme extraction relies on a large LLM (LLaMA) in a largely heuristic, prompt-based manner, yet issues such as output stability, hallucination, and sensitivity to prompt design are not systematically examined.

Thank you for this valuable point. We agree that the current prompt-based approach lacks a systematic examination of output stability. We prioritized empirical validation against expert-labeled ground truth for this study. To rigorously evaluate stability, we would need to approximate the expected output via Monte Carlo sampling. This scales the computational cost C by a factor of k

samples: $C_{total} \propto k \times T_{inference}$. Given the 70-billion parameter size of our model, running k iterations for every review was computationally prohibitive. We have updated the Limitations section to include this technical justification. We also noted that the model successfully identified 82% of human-labeled themes. This empirical result confirms the utility of the zero-shot prompt despite the absence of variance estimation. These revisions appear in Crimson in the revised manuscript.

> **Reviewer # 1-4:** Finally, while the pipeline is presented as an integrated system, most components are evaluated independently, leaving open questions about error propagation and end-to-end behavior in deployment scenarios.

Thank you for this insightful comment regarding system integration and error propagation. We acknowledge that end-to-end evaluation is critical for deployment scenarios. However, we designed FairCareNLP as a modular system with orthogonal information streams to minimize the mathematical risk of error cascades.

In a strictly serial pipeline, the total probability of system success (P_{total}) degrades according to the product of the success probabilities of individual stages (P_i). This relationship is defined as $P_{total} = \prod_{i=1}^{n} P_i$. As a result, a failure in an upstream component such as NER would corrupt the input for downstream tasks like sentiment analysis.

However, our architecture utilizes parallel processing branches. The Sentiment Analysis, Key Theme Extraction, and NER modules process the raw text input independently. This independence addresses the above issues and ensures that the conditional probability of error in one module (E_A) given an error in another module (E_B) remains approximate to the marginal error probability: $P(E_A|E_B) \approx P(E_A)$.

To clarify this, we have updated the **Methods** section to explicitly describe the architectural independence of our approach. We also revised the **Future Directions** section to emphasize the necessity of holistic deployment testing to identify unforeseen system-level interactions. Moreover,we revised **Figure 3** to explicitly highlight the parallel nature of the downstream tasks. These revisions appear Crimson in the revised manuscript.

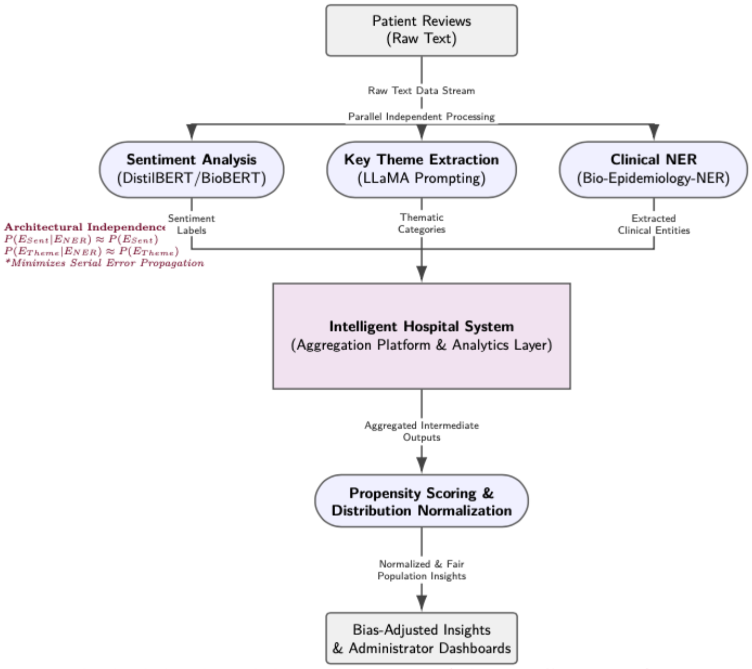

Fig 3. The high-level modular architecture of the FairCareNLP framework. The pipeline integrates distinct stages for preprocessing, debiasing, downstream tasks, and evaluation. While debiasing methods are applied upstream, the downstream models for sentiment analysis and keyphrase extraction operate as parallel, independent processing streams. This design choice ensures that predictive errors within one downstream module do not propagate to parallel tasks.

> **Reviewer # 1-5:** Overall, the paper represents a solid applied contribution and a useful case study on fairness-aware healthcare NLP, but it would benefit from stronger validation, deeper analysis of LLM reliability, and clearer justification of when such a complex multi-module architecture is preferable to simpler alternatives.

Thank you for this important observation regarding reproducibility and practical feasibility. We agree that documenting computational resources and processing times is essential for evaluating the proposed framework. We have updated the manuscript to provide precise details on the hardware infrastructure used for training and inference. We also included estimates of average training duration and end-to-end inference latency per review based on this hardware. These technical specifications are now included in the Experimental Setup section of the revised manuscript in Crimson

Response To Reviewer #2

Reviewer # 2-1: The paper presents an useful attempt toward achieving a more equitable AI in healthcare by showing that bias mitigation techniques (particularly INLP) can improve fairness without substantially sacrificing accuracy, enabling more inclusive patient-centered care. Existing NLP models contain biases based skewing results and causing underrepresentation of certain patient groups, thus perpetuating existing healthcare disparities and compromises patient care. Authors provide sufficient evidence to substantiate the relevance of this issue. In order to mitigate the problem, they propose a comprehensive NLP pipeline that integrates multiple debiasing techniques (adversarial, Hard Debiasing, INLP) rather than using one in isolation, addresses bias at different stages (in-processing and post-processing), applies to multiple demographic attributes simultaneously, and uses propensity scoring to handle self-selection bias. They test their proposed approach using a dataset of anonymized patient reviews together with synthetic hospital-based demographic attributes.

The validation method demonstrates several notable strengths through its comprehensive and systematic approach. The study employs a large-scale real-world dataset of over 120,000 authentic, anonymized, patient reviews from 45 hospitals, providing substantial empirical grounding. The experimental design is rigorous, testing four different BERT models across multiple hyperparameter configurations (three learning rates, three debiasing techniques, varying loss weights), enabling systematic comparison of fairness-performance trade-offs. Critically, the evaluation framework is multi-dimensional, assessing both traditional performance metrics (accuracy, F1, precision, recall, AUC) and fairness metrics (Equalized Odds Difference, Word Embedding Association Test) across multiple protected attributes (gender, ethnicity, socioeconomic status). The study represents a novel integration of three complementary debiasing strategies applied at different pipeline stages, moving beyond prior work that examined single techniques in isolation. Additionally, the propensity scoring module systematically addresses self-selection bias in online reviews with quantitative validation, and the commitment to reproducibility through public data and code sharing strengthens the contribution.

We appreciate your positive assessment of our discussion of the trade offs between fairness and predictive performance. Transparency about the practical challenges of implementing equitable AI in healthcare was a primary motivation for this work. We are encouraged that this aspect of our analysis resonated with you.

Reviewer # 2-2: The most critical weakness undermining the validation is the use of synthetically generated demographic attributes rather than actual patient demographics. These attributes were randomly assigned based on population distributions and (may) have no correlation with the actual review content or writing style, meaning the fairness metrics cannot truly validate whether the models exhibit equitable behavior with real demographic patterns. This fundamental limitation prevents verification of whether bias mitigation actually helps real patients or merely optimizes synthetic benchmarks.

We appreciate this rigorous critique. We agree that synthetic demographics can decouple linguistic features from true societal biases, which limits the ecological validity of the findings. However, this experimental design provides a mechanistic test of the pipeline's debiasing capability. Deep learning models can overfit finite datasets and learn spurious correlations, even from random noise ($I(\hat{Y}; A_{\text{noise}}) > 0$). Our experiments show that the FairCareNLP pipeline neutralizes these algorithmic artifacts by minimizing the mutual information between the embedding space and the sensitive attributes. This result supports the mathematical effectiveness of the INLP and adversarial components in a controlled setting. In addition, synthetic attributes enable us to release a fully reproducible benchmark dataset without compromising patient privacy or violating HIPAA or GDPR requirements. We have updated the Limitations section to clarify the difference between removing linguistic bias and regularizing spurious correlations. These revisions appear in Crimson in the revised manuscript.

Reviewer # 2-3: In considering whether the draft is ready or not for publication, several considerations need to be taken into account. The validation method is methodologically sound for assessing technical performance but fundamentally limited for validating fairness claims due to reliance on synthetic demographics. It successfully demonstrates that certain debiasing techniques (particularly INLP) can maintain model performance while improving fairness metrics on synthetic benchmarks, establishing technical feasibility. However, the disconnect between synthetic demographic labels and actual patient characteristics means the study cannot confirm whether these techniques would produce equitable outcomes for real diverse patient populations. The work represents an important incremental contribution showing how multiple debiasing strategies can be integrated into healthcare NLP pipelines, but it falls short of validating real-world equity impact. To definitely substantiate fairness claims, the research would need either more explicitly acknowledge the limitations, or alternatively, apply additional tests such as requiring validation on datasets with authentic demographic information (using privacy-preserving methods), cross-institutional testing, multilingual evaluation, and prospective studies involving healthcare stakeholders to assess whether the system genuinely supports more inclusive patient-centered care rather than merely optimizing synthetic fairness metrics.

The reviewer fully acknowledges that conducting the alternative tests would necessarily lead to a better substantiated, but also completely different paper. While the research performed is improvable, asking the authors to engage in the work

required to better align with the reviewers' perspective before publication would prevent their unquestionable contribution to immediatly contribute to the discipline, providing a solid and rigorous model for other approaches, and allowing peers to continue this promising line of research. It is necessary to understand that Science is an incremental process made of partial steps. Authors provide a sufficiently solid partial step that others can clearly build upon. Therefore, as long as authors 1) enrich the discussion about privacy preservation, including references to the different approaches (see for example Dwork and colleagues at Harvard on Differential Privacy) and limitations of anonymization (see for example work of Sweeney and associates, also at Harvard), explaining why their chosen approach is the more appropriate one for this paper, and 2) more explicitly state the limitations derived from the use of synthetic demographics, 3) asking for the involvement in future work of social scientists trained in statistical causality (most notably Political Science) as this is the discipline better suited to patch the statistical and causality weaknesses of this paper, then the paper should be considered for publication, and even highlighted in the PONE website.

We are grateful for this constructive roadmap for publication. We agree that, while the current validation demonstrates mechanistic feasibility, the use of synthetic data requires a clear ethical justification related to privacy. We have expanded the **Discussion section** by adding a new subsection, titled Privacy Preservation and Synthetic Benchmarks, to frame the use of synthetic demographics within the context of privacy utility trade-offs. We now explicitly cite the foundational work of **Sweeney and Dwork** to argue that decoupling real demographics from unstructured free-text is essential to prevent re-identification attacks in public benchmarks. Furthermore, we have updated the **Future Directions** section to explicitly call for interdisciplinary collaboration with social scientists and the application of causal inference frameworks to validate these findings on sensitive, non-public clinical data. These revisions appear in Crimson in the revised manuscript.
