# Supplementary material for: FairCareNLP: An AI-driven patient review analyzer for healthcare
Source: PLoS One. 2026 May 4;21(5):e0337676. doi: 10.1371/journal.pone.0337676 (PMC13138667; doi:10.1371/journal.pone.0337676)
Supplement: Attachment — Submitted filename: 2026_Pourya_s_Paper__2_AI-rebuttal.pdf [file pone.0337676.s004.pdf]

Response to the Reviews and Decision

FairCareNLP: An AI-Driven Patient Review Analyzer for Healthcare

Manuscript Reference Number:
PONE-D-25-60609-R1

Date: February 16, 2026

Dr. Issa Atoum
Academic Editor
PLOS One
Submission Date: February 16th, 2026

Subject: Revision and re-submission of manuscript **PONE-D-25-60609R1**

Dear Dr. Issa Atoum,

Thank you for the opportunity to continue revising our paper. We greatly appreciate the constructive feedback from you and the reviewers. We have responded to the additional comments from Reviewer 2.

We look forward to hearing from you and hope that the revised manuscript will better suit PLOS One.

Sincerely,
Zahra Shakeri
Assistant Professor
University of Toronto

Response To Reviewer #2

Reviewer # 2-1: In a prior review, authors have been suggested to appropriately address the sensitive nature of the data. However, in the re-submitted version, this issue remains unaddressed. Provided the sensitive nature of the involved data, and derived high risk, the draft in the current version cannot be considered ready for publication. Authors need to apply effective privacy-preserving techniques capable to ensure that no personal data is leaked. See my prior review with specific details on this issue.

We thank the reviewer for clarifying this comment and for the helpful emphasis on privacy protections for unstructured patient narratives. We agree that free-text patient narratives require strong privacy protection and that masking direct identifiers (e.g., replacing names with "XXXX. . . ") does not, by itself, eliminate re-identification risk. In this revision, we improved the manuscript to (i) explicitly acknowledge residual linkage risk in de-identified narratives, (ii) clearly describe the layered privacy controls used in our study, and (iii) explain how formal differential privacy mechanisms would be incorporated in a deployment-oriented next step.

Manuscript changes: (1) **Rewrote Section 4.4 (Privacy Preservation and Synthetic Benchmarks).** We replaced the prior Section 4.4 with a new discussion including a mock example showing why residual re-identification risk can persist even after "XXXX. . . " masking, and we detail our mitigations, including provider de-risking via manual review, data minimization (e.g., coarse month/year only and no exact visit dates), restricted-access processing, and screened reporting examples. We also clarify the role of synthetic demographics as a fairness benchmarking tool that reduces the need to use true individual-level demographics.

(2) **Clarified LLM handling of narrative text (Sections 2.2.1 and 2.3).** Because narrative text is wrapped into the theme-extraction prompt, we explicitly state that LLaMA inference was performed within our institutional environment and that record-level narrative text was not transmitted to external LLM APIs. We also clarify that any LLM-assisted unit/type normalization did not involve narrative text.

(3) **Revised Data Availability.** We updated Data Availability to remove ambiguity about releasing record-level narratives. The public repository contains code and non-identifying derived artifacts sufficient to reproduce reported results, while raw narrative text is not redistributed due to residual re-identification risk inherent to unstructured text.

(4) **Formal privacy mechanisms as future deployment work (Section 4.4).** We added a concise explanation of differential privacy (DP) and an illustrative example (i.e., DP release of theme counts) to clarify how DP would be applied to derived releases in future deployments. This study does not claim a DP guarantee and suggests DP integration for deployment when releasing aggregates or model checkpoints.

Below are screenshots of the changes we made, highlighted for clarity. All edits are highlighted throughout the revised manuscript.

2 Materials and methods

2.1 Dataset

This study uses a dataset from patient surveys administered by the National Research Corporation (NRC), a US-based organization located in Ontario, Canada. The surveys include quantitative metrics and open-ended responses about hospital experiences, and they provide self-reported patient feedback. The Investigative Journalism Bureau (IJB), affiliated with the Dalla Lana School of Public Health at the University of Toronto, acquired the de-identified dataset covering five years through freedom of information requests. The resulting compilation includes more than 120,000 anonymized patient reviews from 45 hospitals in Ontario. These reviews span 2015 to 2022. Our dataset consists of free-text patient experience comments associated with hospital encounters (e.g., inpatient vs. emergency) and coarse time information (month/year). We intentionally discard exact visit dates and retain only month/year granularity to reduce linkage risk. Because these narratives may contain sensitive health information, we treated the dataset as sensitive throughout the project even when direct identifiers were not present. Prior to analysis, the text records were de-identified by the data provider via manual review, with direct identifiers removed or replaced by placeholder tokens (e.g., 'XXXX...') in the free-text field. Direct identifiers targeted for removal included (but were not limited to) personal names, clinician/staff names, addresses, email addresses, phone numbers, patient identifiers, and other explicit contact/location details. This de-identification was performed through a dedicated manual review process prior to our receiving the data. We did not attempt to re-identify any individual, and no linkage to external datasets was performed. Ontario hospitals and health networks distributed these surveys, which captured various aspects such as hospital attributes (e.g., name, type, units), patient experience (e.g., patient reviews, visit date, sentiment valence), and key themes affecting patient satisfaction (e.g., respect, transportation, access, coordination). The dataset has been annotated to allow for the possibility of multiple key themes being associated with each patient review. Analysis were completed

---

2.3 Key Theme Analysis Module

After processing the review and extracting sentiments, we have our key theme analysis module. For key theme analysis, we engineered a prompt and wrapped the reviews with that prompt, using LLaMA3 70B to identify the key themes and the corresponding sections in the reviews for those key themes. All LLaMA inference was performed within our institutional compute environment and record-level patient narrative text was not transmitted to external LLM APIs. We used the following prompt:

```
prompt = ( "You are analyzing a patient review to identify key themes or areas
discussed in the text.  "
"Key themes are specific topics, concerns, or aspects of the healthcare experience
that the patient "
"mentions or talks about in their review.\n\n"
"Analyze the following patient review and identify all key themes from this list:
```

4.4 Privacy Preservation and Synthetic Benchmarks

Unstructured patient experience narratives are inherently sensitive. Even after removal of *direct identifiers* (e.g., names) using placeholder tokens such as "XXXX…", there can remain a *residual* risk of re-identification via contextual uniqueness and linkage to outside knowledge (i.e., linkage attacks) [56]. This risk is particularly relevant when free-text narratives are combined with granular metadata (e.g., exact dates, fine geography, or true demographics). For example, consider a single record with fields consistent with our dataset structure:

$$r_i = (h_i, u_i, t_i, \text{month/year}_i),$$

where h_i is a hospital, u_i is a unit (e.g., emergency or inpatient), t_i is free text, and month/year_i is the coarse visit time. A narrative may be de-identified as *"Dr. XXXX explained my results after a rare complication during my pregnancy; my partner could not accompany me due to pandemic restrictions"*. Even though direct identifiers are masked, the combination of (i) a rare event, (ii) a specific hospital/unit, and (iii) time period can still be potentially linkable by an adversary with external knowledge. We therefore treat the corpus as sensitive throughout the workflow and do not interpret "XXXX…" replacement as a complete privacy solution on its own.

To address this, we implemented a layered privacy approach designed to materially reduce disclosure risk for unstructured text:

- **Provider de-risking/de-identification.** The dataset was received in de-identified form. The data provider performed manual review using a dedicated process (and personnel) to remove or replace direct identifiers in the narrative text (e.g., personal names, staff names, contact/location details). This process produced placeholders such as "XXXX…".
- **Data minimization.** We intentionally limit metadata granularity. We keep only coarse time fields at the month and year level and do not use exact visit dates in modeling or reporting. We further avoid introducing true individual-level demographics. Instead, we use *synthetic* demographic attributes for fairness benchmarking.
- **Restricted-access processing and screened reporting.** Record-level narratives are processed in a controlled environment with access limited to authorized study personnel, and we do not attempt to re-identify individuals or link to external datasets. To reduce risk in dissemination, the illustrative narrative examples included in this manuscript were reviewed to confirm that no direct identifiers are present.

The protections above focus on de-identification, minimization, governance, and disclosure control. A complementary direction for future deployment is to incorporate *formal* privacy guarantees for *derived releases* (e.g., aggregate statistics, dashboards, or released model checkpoints) using differential privacy (DP) [57]. Given that the dataset

Data Availability

The public repository contains code and analysis scripts needed to reproduce the reported figures and tables. Due to the sensitive nature of record-level free-text patient narratives and residual re-identification risk inherent to unstructured text, we do not redistribute the raw comment text in the public repository. Where possible, we provide derived non-identifying artifacts (e.g., aggregate statistics and evaluation outputs) sufficient to reproduce the main results. The code repository is available at https://github.com/HIVE-UofT/Patient-Review-Analyzer.
